# Mimetic Neural Networks: A Unified Framework for Protein Design and Folding

**DOI:** 10.3389/fbinf.2022.715006

**Published:** 2022-05-05

**Authors:** Moshe Eliasof, Tue Boesen, Eldad Haber, Chen Keasar, Eran Treister

**Affiliations:** ^1^ Department of Computer Science, Ben-Gurion University of the Negev, Beer-Sheva, Israel; ^2^ Department of EOAS, The University of British Columbia, Vancouver, BC, Canada

**Keywords:** graph neural networks, protein design, protein folding, deep learning, protein sructure prediction

## Abstract

Recent advancements in machine learning techniques for protein structure prediction motivate better results in its inverse problem–protein design. In this work we introduce a new graph mimetic neural network, MimNet, and show that it is possible to build a reversible architecture that solves the structure and design problems in tandem, allowing to improve protein backbone design when the structure is better estimated. We use the ProteinNet data set and show that the state of the art results in protein design can be met and even improved, given recent architectures for protein folding.

## 1 Introduction

Protein structure prediction (***PSP***) and protein backbone design are two related tasks. The former, aims at deriving three dimensional models of proteins from their residue (amino-acid) sequences. The latter, protein backbone design, aka the inverse protein folding problem, focus on suggesting sequences that are likely to fold to a given structure. These two scientific problems have remained open for more than 4 decades already, with unprecedented recent successes (Sun and Kim, 2017; Strokach et al., 2020; Ben-Sasson et al., 2021; Jumper et al., 2021). They have common underlying physical principles, and consequently the same computational infrastructure may cope with both (Jones, 1994; Leaver-Fay et al., 2011; Norn et al., 2021). Yet, these problems are typically considered separately, with many scientific endeavours focusing on either prediction or design but not on both. Thus, the current breakthrough in PSP does not directly affect the design filed, which seems to somewhat lag behind. Here we present a unifying framework that handles both problems simultaneously offering a conceptual way to harness progress in PSP to push the design field forward. Our method, dubbed MimNet, is inspired by the reversibility of the folding/unfolding physical processes, and the ability of molecular dynamics simulations to reproduce this behaviour. We use essentially the same mathematical formulation to mimic (hence the name) the physical processes by non-physical, alchemy, processes that reversibly transform “coordinates” between sequence and Cartesian spaces. To this end we leverage bi-directional Graph Convolutional Neural Networks (GCNs) that solve both problems, PSP and design, using the same learned weights.

Evolutionary inference, mainly in the form of multiple sequence alignments (MSAs) of homologous proteins, has long been recognized as a major source of structural insight. First order statistics of MSAs including conservation profiles, position-specific score matrices (PSSMs) (Gribskov et al., 1987; Altschul et al., 1997) and hidden Markov models (Remmert et al., 2012) allow the identification of remote homologs as modeling templates (Bates et al., 2001; Schwede et al., 2003; Hildebrand et al., 2009; Waterhouse et al., 2018), and reliable prediction of one-dimensional structural features like secondary structure (Rost and Sander, 1993), transmembrane segments (Rost et al., 1995), and solvent accessibility (Adamczak et al., 2004). Second order statistics, like direct coupling (Morcos et al., 2011) and inverted covariance matrices (Jones et al., 2012; Li et al., 2019), are used to predict spatial contacts between residues (Vassura et al., 2008; Kamisetty et al., 2013; Tetchner et al., 2014). The use of second order statistics require “deep” MSAs, that is alignments of many diverged homologous sequences. Searching for these homologous sequences, in large and rapidly growing datasets, becomes a major task in PSP that consumes much time and computational resources. Further, the sheer size of “deep” MSA datasets makes them hard to share.

In recent years, deep learning techniques boost the efforts to solve the PSP problem (AlQuraishi, 2019a; Drori et al., 2019; Hou et al., 2019; Kandathil et al., 2019; Xu, 2019; Zheng et al., 2019; Senior et al., 2020; Baek et al., 2021), reaching remarkable unprecedented performance, as was demonstrated in the CASP13 and CASP14 competitions (Abriata et al., 2019; Kryshtafovych et al., 2019; Jumper et al., 2021). Currently, all the state-of-the-art, high performance, methods rely on second order statistics derived from “deep” MSAs. In this study we chose an alternative rout to PSP, namely using first order information only (Li et al., 2017; Gao et al., 2018; AlQuraishi, 2019a; Torrisi et al., 2020; Xu et al., 2021). Giving up on the structural clues provided by second order information, this approach has received less attention in recent literature. Nevertheless, by using only first-order statistics we are able to focus on the development of new prediction strategies with reasonable resources. To this end, we follow the example of (AlQuraishi, 2019a) and benchmark on the relatively light-weight ProteinNet (AlQuraishi, 2019b), in which the only inputs to PSP are sequences and PSSMs (formulated as a per-position probability density function). We further adopt a lean protein representation with a single interaction point, the *Cα* atom, per residue. Finer grained protein representations (i.e., more interaction points per residue) are more expressive, and thus likely to achieve better results. Yet, when developing a novel methodology we do not aim at state-of-art performance but rather at simplicity and ease of experimentation. The *Cα* representation for that matter is expressive enough. Yet, our MimNet is on-par with previous studies that used only first order data, and it outperforms (AlQuraishi, 2019a), which uses the same training and test sets, and thus can be directly compared (see Section 3.3).

PSP gained much more attention over the years compared with its inverse, protein design. One apparent reason, for the more central role of PSP, is that progress in this field is monitored and accelerated by two accepted and objective benchmarks: Critical Assessment of Structure Prediction (***CASP***) (Moult et al., 1997; Kryshtafovych et al., 2019) and Continuous Automated Model EvalutiOn (CAMEO) (Haas et al., 2018). The protein design field lacks benchmarks like CASP and CAMEO, and a reliable evidence of success requires expensive synthesis and functional characterization in the laboratory (Dahiyat and Mayo, 1996; Basanta et al., 2020; Ben-Sasson et al., 2021). When laboratory validation is infeasible (e.g., this study), researchers resort to re-designing proteins of known structures.

Notwithstanding the evident importance of CASP and CAMEO they compare the “end product” (i.e., prediction) of elaborate pipelines. A mediocre implementation of one stage of the pipeline may overshadow other, better implemented stages. Most importantly, data collection and preprocessing may dominate the performance of data driven methods. The PSP data-set of this study, ProteinNet, offers an alternative type of benchmark. In the spirit of machine learning data-sets such as ImageNet (Deng et al., 2009), it provides predefined training and test sets (based on the PDB and on CASP validation) and allows researchers to focus on the development of novel machine learning schemes. Using a reversible network, this study begs to use the same data-set also as a benchmark for its design side. Specifically, we aim to reproduce the residue probabilities of ProteinNet’s PSSMs based on the *Cα* Coordinates. Indeed, a network can learn to generate sequences (Strokach et al., 2020) as the “natural” output of a design algorithm. However, sequences are in a sense arbitrary, as homologous proteins share similar if not identical (up to measurement errors) structures, while their sequences are diverged. Thus, the design problem of some structure does not have a unique solution, but rather a family of sequences that we represent by a PSSM that associates each position with a residue probability distribution function. Individual sequences may then be sampled form the PSSM or, if the target structure exists in nature, retrieved from sequence data-sets with a search algorithm (e.g., BLAST). The former is similar in spirit to the veteran back-to-consensus strategy of more traditional protein design approaches (Bershtein et al., 2008; Chandler et al., 2020).

Employing deep learning for protein design task is a relatively new idea (O’Connell et al., 2018; Ingraham et al., 2019; Anand-Achim et al., 2021; Norn et al., 2021). A similar approach to ours was recently presented in (Strokach et al., 2020) where graph methods were proposed for protein design, reporting promising results by treating the problem as a graph node classification problem, surpassing other *de-novo* design codes. While (Strokach et al., 2020) and our method share some similarities, ours is largely different as we use reversible architectures which offer numerous advantages, discussed in the following. As we show in Section 3.2, our approach obtains better results on a large data-set derived from the Protein Data Bank (PDB).The main part of this work is the introduction of a framework that unifies the treatment of protein folding and design. Our framework mimics the physical formulation of protein folding using a neural network. Hence, we coin the term *Mimetic Deep Neural Networks* (MimNet), which we apply to graphs, describing protein structures. While our work focuses on protein folding and design, the proposed network can be applied with any node or edge data that is available, thus it suits using both first or second order statistics.

The main idea is to generate a reversible transformation from the structure to the sequence and vice versa by using reversible neural network architectures (Chang et al., 2018; Ruthotto and Haber, 2019). These networks are bi-directional (can propagate forward and backward), and hence allow us to jointly train them to solve both the folding and the design problems, utilizing both the sequence and the structure of the protein *simultaneously*. This effectively doubles the amount of the data with respect to the network parameters. Such networks can utilize any type of layer, from structured to graph convolution or attention, harnessing recent advances in the understanding of protein folding architectures. Another important advantage of such a network is its memory footprint. Since the network is reversible, it is possible to train an *arbitrarily long* network without storing the activations, at the cost of double the computation of the backward pass (Chang et al., 2018). This enables the use of very deep networks that are impossible to use otherwise.

Furthermore, the physical folding process can be described by a second order differential equation derived from Hamiltonian dynamics. Therefore, we consider reversible architectures that are inspired by Hamiltonian dynamics hyperbolic differential equations (Chang et al., 2018; Ruthotto and Haber, 2019), which can be used to simulate this process. One can therefore claim that such a mimetic network is more faithful to the physics of the protein folding problem compared to a standard deep network like a ResNet (He et al., 2016). In particular, using such a dynamics has conservation properties that avoid the well known phenomenon of over-smoothing in graphs (Ming Chen et al., 2020; Zhao and Akoglu, 2020; Chamberlain et al., 2021; Eliasof et al., 2021). In this paper, we particularly explore the use of reversible GCNs, which are graph-based deep learning methods (Gao and Ji, 2019; Wang et al., 2019). Such networks are inspired by molecular dynamics, and their pairwise interactions are reminiscent of the residue interactions in a three dimensional physical system.

The rest of the paper is organized as follows. In Section 2 we discuss the problem and introduce the key mathematical ideas which constitute the building blocks of our network. In particular, we discuss multiscale reversible networks and different types of graph convolution techniques that are used to solve the problem. We then define our MimNet and its objective functions. In Section 3 we perform numerical experiments with data obtained from ProteinNet (AlQuraishi, 2019b). ProteinNet is a publicly available data set that contains both sequences and PSSMs and thus allows for the training of a folding network with first order statistics as done in the recurrent geometric network (***RGN***) (AlQuraishi, 2019a). The size of the data set, its structure, and the division into training validating and testing subsets, were carefully selected, allowing one to rigorously test the design problem as well. Finally, in Section 4 we discuss the results and summarize the paper.

## 2 Methods

Before discussing the particular network and architecture, we define the data and the functions of folding and design problems. Specifically, assume that S∈S is a 20, ×, *n* matrix that represents a protein sequence of *n* amino acids. Let $S^{+}\in S^{+}$ be additional data that is related to the sequence such as PSSM and possibly covariance information derived from MSAs. Also, let X∈X be a 3 × *n* matrix that represents the protein structure (coordinates). We define the mapping $F:S\times S^{+}\to X$ as the folding mapping. This mapping takes the information in **S** and **S** ^+^ and maps it into the estimated coordinates $\hat{X}$ that reveal the structure of the protein. Throughout the paper we denote *F*(**S**) instead of *F*(**S**, **S** ^+^) for brevity. Consider now the opposite mapping from the space X to the space $S\times S^{+}$ . We denote this mapping as $F^{†}:X\to S\times S^{+}$ and it can be thought of as some psedu-inverse of the mapping *F*. These mappings can be learnt separately and independently as has been done so far. However, since *F* and *F* ^†^ are closely related, it is tempting to jointly learn them, utilizing both the sequence, its attributes, as well as the structure of the protein *in tandem*.

We now review the concept of a mimetic deep neural network, that is, a neural network whose functional form mimics simulations of folding dynamics. To this end, a deep network can be thought of as a time discretization of a differential equation (Chen et al., 2018; Ruthotto and Haber, 2019). According to this interpretation, each layer represents the state of the system at some particular pseudo-time. The mimetic properties are first discussed in pseudo-time, namely, how the network propagates from one layer to the other. The second mimetic property considers the spatial domain, meaning, how a particular residue in the protein interacts with another residue. These properties are put together to generate a mimetic deep neural network that imitates Verlet integration in a high dimensional space, using network architectures that are derived by discretized differential operators in time and space (Ruthotto and Haber, 2019; Eliasof and Treister, 2020). The treatment in both space and time are put together within a network optimization procedure to train the system and yield a network that can solve both the folding and the design problems.

### 2.1 Reversible Networks and Dynamical Systems

In this subsection we show how to build a mimetic network in time by using reversible dynamics. Reversible systems play a major role in physics for applications that range from Hamiltonian dynamics to wave equations. Broadly speaking, a reversible system is one that can propagate forward in time without information loss and therefore, can propagate backwards in time. Simple physical examples are a pendulum or a wave. These systems (in their idealized form) do not change their entropy, and therefore allow for forward or backward integration in time. Typical molecular dynamics is solved using reversible methods (Allen, 2004) (that is, integrating Hamiltonian dynamics) and therefore, it is natural to explore neural network architectures with similar properties.

To be more specific, given the input for the folding task [**S**, **S** ^+^] (e.g., the concatenation of the one hot encoding sequence design and PSSM matrices) we first apply

$$Y_{0}=q\left(\left[S,S^{+}\right],\theta_{e}\right)$$ (2.1)

where **Y**_0_ contains *n*_*f*_ channels of *n*-length sequence features, embedded by the transformation *q*(⋅, ⋅), parameterized by the weights ***θ***_*e*_. This layer transforms the input to the latent space of the network. Here we use a 1D convolution for *q*, but other transformations may also be suitable.

The initial state **Y**_0_ and its velocity vector **V**_0_ are then pushed forward by a deep residual neural network. In particular, we consider a network with the following structure

$$\begin{array}{ll} V_{j+1} & =V_{j}+h\cdot f\left(Y_{j},\theta_{j}\right) \end{array}$$ (2.2a)

$$\begin{array}{ll} Y_{j+1} & =Y_{j}+h\cdot g\left(V_{j+1},\theta_{j}\right), \end{array}$$ (2.2b)

where *j* = 0, … , *T* is the layer index. *h* is a parameter that represents a time step size and ***θ***_*j*_ are learnt parameters that characterize the *j*-th layer. The system in Eq. 2.2a can be interpreted as a Verlet type discretization of a dynamical system with learnable forces that are the gradients of some potential function. A particular case of such dynamics is obtained by setting *g* = *Id* (the identity transformation) yielding the second order dynamics

$$Y_{j+1}=2Y_{j}-Y_{j-1}+h^{2}f\left(Y_{j},\theta_{j}\right).$$ (2.3)

**FIGURE 1:**
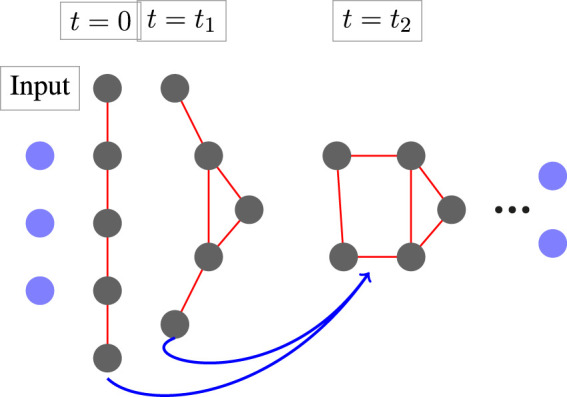
The architecture of MimNet with graph convolution layers. An embedding layer transforms the input into a latent space which then propagates through a GCN layer fed with the outputs of two previous layers. A graph that represents the protein structure is computed after each layer. The final layer is then projected back to obtain residue coordinates.

This scheme is reversible, regardless of the choice *f* (which we discuss Section 2.3), since we can express **Y**_*j*−1_ as a function of **Y**_*j*_ and **Y**_*j*+1_. The propagation forward (and backward) is not complete without defining the boundary conditions **Y**_−1_ and **Y**_*T*+1_. Here we arbitrarily choose **Y**_−1_ = **Y**_0_ and **Y**_*T*−1_ = **Y**_*T*_, that is, initializing the network with zero velocity, i.e., **V**_0_ = 0. An illustration of the dynamics is plotted in Figure 1. Note, that this formulation is a slight (but important) modification of the standard ResNet (He et al., 2016) that reads

$$Y_{j+1}=Y_{j}+hf\left(Y_{j},\theta_{j}\right).$$ (2.4)

Given the final state of the system **Y**_*T*_, we predict the coordinates **X** by projecting **Y**_*T*_ onto a three dimensional space

$$\hat{X}=q^{+}\left(Y_{T},\theta_{f}\right),$$ (2.5)

where $\hat{X}$ are the predicted coordinates. The transformation *q* ^+^(⋅, ⋅) can be realized by a neural network, and we choose it to be a learnable projection matrix of size *n*_*f*_ × 3 such that the final feature maps are projected to 3D coordinates. The layer in Eq. 2.5 may also contains some additional constraints. In particular, we may demand that

$$|{\hat{X}}_{i}-{\hat{X}}_{i-1}|=c,$$

constraining the distance between every two residues to *c* = 3.8 Å. We have found that when the data is noisy implementing this constraint is needed in order to obtain physically feasible results (see Section 2.5.3).

In the forward pass, described above, the folding problem was solved, where we march from the protein design attributes (as in Section 2.1) to its coordinates. In the backward pass, we solve the design problem, where our goal is to predict the sequence given its coordinates. We start the backward pass by embedding the coordinates into the network feature space, i.e.,

$$Y_{T}=\left(q^{+}\right)*\left(X,\theta_{f}\right),$$ (2.6)

where $\left(q^{+}\right)*$ is the adjoint of the transformation *q* ^+^. We then march backwards, replacing the entries of **Y**_*j*+1_ and **Y**_*j*−1_ in Eq. 2.3 and finally, using the adjoint of *q* to propagate from **Y**_0_ to the sequence space

$$\left[\hat{S},{\hat{S}}^{+}\right]=q*\left(Y_{0},\theta_{e}\right),$$ (2.7)

where ${\hat{S}}^{+}$ is the predicted PSSM, and $\hat{S}$ is a one-hot representation of the predicted sequence (the top probability residue-type in each position). These forward and backward passes couple the design and the folding tasks together into a single network that, similarly to the physical dynamics, can be integrated (in time) from sequence to coordinates and backwards from coordinates to a sequence.

### 2.2 Graph Convolutional Networks

Section 2.1 considers the propagation of the network from its initial condition (a sequence) to its final one (3D structure) and vice versa. The discussion was agnostic to the choice of the function *f*(⋅, ⋅) in Eq. 2.3 that realizes the network in hand. In this section we review the concept of a graph network and discuss its computation.

The idea behind a graph based method is rooted in the physics of the problem. Energy based simulations can be thought of as pairwise interactions on a graph based on the *L*_2_ distance between the residues. Indeed, as the distance between residues is smaller, the interaction between them is stronger. This motivates us to use machine learning techniques that mimic this property. As the dynamical system is evolving, the interactions between pairs of close residues is significantly larger compared to far ones.

One of the most successful techniques for image and speech processing is Convolution Neural Networks (CNN) (Krizhevsky et al., 2012; Goodfellow et al., 2016). The method relays on the structured grids on which sequences and images are defined. That is, every element has neighbouring elements in a structured manner. In recent years, similar ideas were extended to more complex geometries and manifolds, which can be conveniently represented by a graph (Ranjan et al., 2018; Hanocka et al., 2019; Wang et al., 2019). The main idea is to replace the structured convolution with a graph based convolution. That is, rather than convolving each location in the feature map with its near neighbours, define the distance between each location based on node or edge features, and then convolve the residues that are close on the graph.

To be more specific, we let **Y**_*j*_ be the state at the *j*th layer. Then, we define a graph convolution block as follows:

$$f\left(Y_{j}\right)=-C^{*}\left(\theta_{j},\sigma \left(C\left(\theta_{j},Y_{j}\right)\right)\right)$$ (2.8)

where $C\left(\theta_{j},\cdot \right)$ is the graph convolution operator with its learned associated weights *θ*_*j*_. This operator spatially resembles a discrete differential operator, e.g., a mass term, a graph Laplacian, or an edge gradient (Eliasof and Treister, 2020). *σ*(⋅) is the ReLU activation function. The operator $C^{*}$ is the adjoint operator of C (like a transposed convolution), applied using the same weights *θ*_*j*_. This way, assuming that *σ* is a monotonically non-decreasing function that either zeroes its input or preserves its sign, we get a symmetric and positive semi-definite operator. We use the negative sign in front of the layer such that the operator *f*(⋅) is negative, which is important if we are to generate a stable dynamics—see (Ruthotto and Haber, 2019) for details and analysis.

Many graph based networks employ a graph convolution with fixed connectivity (Ranjan et al., 2018; Bouritsas et al., 2019). This is reasonable if the final topology is known. However, for protein folding we start with an unknown structure and it is evolving (learnt) from the data. Therefore, rather than using a fixed graph for the network we let the graph evolve throughout network. We thus recompute a weighted graph Laplacian at each layer, or, for computational saving, every few layers. To this end, we compute the weighted distance matrix between each two residues

$$W_{j}=\exp\left(-\alpha^{-1}D\left(Y_{j}\right)\right),$$ (2.9)

where *α* is a scaling parameter (we set *α* = 10) and **D** is the *L*_2_ distance between each two residues

$$D\left(Y\right)=\left(Y^{2}11^{⊤}+11^{⊤}Y^{2}-2Y^{⊤}Y\right).$$ (2.10)

The vector **1** is a vector of ones of appropriate size. Using the distance matrix we define the graph Laplacian as

$$L_{j}=\mathrm{diag}\left(D_{j}1\right)-D_{j}.$$ (2.11)

The approach of dynamically updating the connectivity of the graph was also suggested in (Wang et al., 2019), where *k* nearest neighbors are equally chosen per node, regardless of their distances. However, this imposes a non-smooth transition of the graph, which may result in optimization difficulties. Thus, we employ a weighted fully connected graph that can smoothly strengthen or weaken the connectivity of the residues. Since the weighting of the edges makes the graph Laplacian continuously differentiable with respect to the network parameter - a smoother optimization trajectory is obtained, leading to better results in our experience.

### 2.3 Multiscale Graph Networks

The limitation of graph based networks, similar to other convolution methods is that they generate strong local interactions only. Hence, spatially-distant connections may suffer from weak interactions (due to small weights), and information will be spread slowly within the network—requiring more layers to compensate for. An elegant way to introduce long-range interactions and pass information between far-away parts of the graph is to consider a multiscale framework.

To this end, instead of a standard graph convolution C in Eq. 2.8, we use a multiscale mechanism that is similar to a U-net (Ronneberger et al., 2015; Shah et al., 2018), where coarse scale approximations of the protein are composed. In particular, in the multiscale version of Eq. 2.8 we choose C in to be the encoder part of a U-net, and the operator $C^{*}$ is the transposed operation, that has a decoder structure (parameterized by the same weights). Together, they form a symmetric graph U-net. The reversibility of the networks remains, since Eq. 2.3 is reversible for every *f*, and in particular for our symmetric U-net.

**FIGURE 2:**
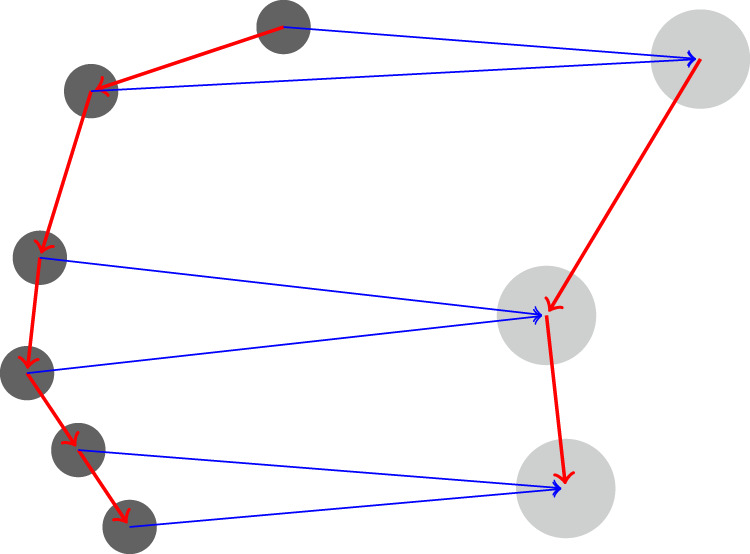
Coarsening a protein. The features of each two residues in the chain (left graph) are averaged together and a new coarse graph is computed (right) for the coarse protein. The graph Laplacian is computed directly from the rediscretized coarse protein.

**FIGURE 3:**
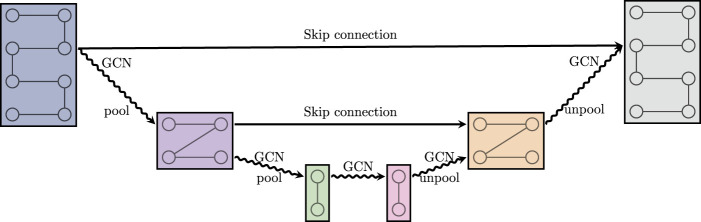
A graph U-net. GCN is defined in (2.12). Pool and unpool denote graph coarsening and refinement, respectively. Skip connection denotes a summation of the respective feature maps.

Our graph U-net is comprised of *n*_*Levels*_ graph scales. At each level we perform a GCN block where we use both the graph and sequence neighbors in our convolutions:

$$Y_{j+1}=\omega_{j}Y_{j}+\sigma \left(N\left(K_{j}\left(Y_{j}+Y_{j}L_{j}\right)\right)\right).$$ (2.12)

where **K**_*j*_ is a 1D convolution with kernel of size 9, connecting nodes on the protein sequence, and **L**_*j*_ is the graph Laplacian operator from Eq. 2.11. N is the instance normalization layer, and *σ* is the ReLU activation. *ω*_*j*_ equals 1 when graph coarsening is not applied, and 0 otherwise. On the coarsest level of the U-net, we perform two convolution steps like Eq. 2.12. At each level, the graph differential operator **L**_*j*_ is re-computed on the coarse graph allowing for simple and inexpensive computations between scales. In addition, since the protein has a simple linear underlying chain, we use linear coarsening, implemented by simple average 1D pooling—this is illustrated in Figure 2. In the decoder part of the U-net we apply the transposed operators, and to refine our graph (unpooling) we use a linear interpolation along the chain. To propagate information between matching levels we add long skip-connections after each convolution, for a stable training scheme (see Figure 3). For the U-net depth, we chose *n*_*Levels*_ = 3, which provides a good trade-off between performance and computational requirements.

### 2.4 The MimNet Architecture

Combining our building blocks together, we now define our bi-directional mimetic architecture, called MimNet. The network consists of three main components - the opening embedding layer, a stacked graph U-net modules, and a closing embedding layer.

At the start and end of MimNet we use the embedding layers Eq. 2.1 and Eq. 2.5, both of which are implemented using a simple 1 × 1 convolution of appropriate sizes (*n* = 40). At the core of our network we employ a series of *T* graph U-nets modules. Each graph U-net is defined according to section 2.3, and all of them are of identical dimensions. That is, each has *n*_*f*_ channels of equal dimensions on the finest level.

### 2.5 Training MimNet

Our MimNet allows us to build the physics of the problem into the neural network. This needs to be followed by a thoroughly thought training process. In particular, care needs be taken when choosing the appropriate problem to minimize and the appropriate choice of loss functions and regularization. We now discuss these choices for our training.

#### 2.5.1 The Optimization Problem

Since we have a bi-directional network, we use both directions to train the network. We define the objective function

$$J\left(\theta \right)=\frac{1}{N}\underset{j}{\sum }\ell_{fold}\left(F\left(S_{j},\theta \right),X_{j}\right)+\ell_{design}\left(F^{†}\left(X_{j},\theta \right),S_{j}\right)+\beta R\left(\theta \right).$$ (2.13)

Here ***θ*** are all the parameters of the network, *F* is the forward network from sequence to coordinates and *F* ^†^ is the backward mapping from coordinates to sequence. The loss functions *ℓ*_*fold*_ and *ℓ*_*design*_ are chosen to measure the discrepancy between the estimated and true coordinates and between the predicted and true sequence design, respectively. The choice of these functions is to be discussed next. Finally, *R*(⋅) is a regularization term that ensures stability of the network and is described below.

#### 2.5.2 Loss Function for the Design Problem

The loss function for the design problem aims at an accurate PSSM and sequence prediction by the network. Noting

$$\left[\hat{S},{\hat{S}}^{+}\right]=F^{†}\left(X_{j},\theta \right).$$ (2.14)

We interpret both $\hat{S}$ and ${\hat{S}}^{+}$ as matrices for which their *ij*-th entry is the probability of the *j*-th residue to be of residue-type *i*. Thus, a natural comparison between two PSSMs is the KL-divergence Silverman (1986) that compares the predicted and ground-truth distributions, setting

$$\ell_{\text{design}}=\sum S^{+}⊙\log\left({\hat{S}}^{+}⊘S^{+}\right)+\sum S⊙\log\left(\hat{S}⊘S\right),$$ (2.15)

where ⊘ denotes element-wise division. Having a PSSM as an output allows for greater flexibility when designing a protein, since there is not necessarily a unique answer to the design process, and the PSSM represents this ambiguity. As we show later, utilizing the PSSM-based loss in Eq. 2.15 has a significant contribution to the accuracy of the network compared to a loss based on the sequence alone used in (Strokach et al., 2020). Thus, as we demonstrate later, the coupling of PSSM and sequence estimation yields favorable performance.

#### 2.5.3 Loss Function for the Folding Problem

We turn our attention for the loss function for the folding problem. Clearly, one cannot simply compare the coordinates obtained by the network, *F*(**S**) to the observed coordinates of the sequence, as they are invariant with respect to rotation and translation. Similar to the work (AlQuraishi, 2019a) one can compare the distance matrices obtained from the coordinates. Let $D_{s}\left(X\right)=\sqrt{D\left(X\right)}$ be the pairwise distance matrix in Eq. 2.10. The distance matrix is invariant to rotations and translations. Thus, it is possible to compare the distances obtained from the true coordinates, **D**_*s*_(**X**) to the distances of the predicted coordinates **D**_*s*_(*F* ^†^(**S**)) by their dRMSD in Eq. 2.16.

$$\ell_{fold}=\sqrt{\frac{1}{n_{M}}{\left\|M⊙\left(D\left(F\left(S,\theta \right)\right)-D\left(X\right)\right)\right\|}_{F}^{2}}$$ (2.16)

Where **M** is a masking matrix, and *n*_*M*_ is the number of non-zeros in **M**. Zero elements in **M** correspond to either missing data in the native structure or to large distances. In proteins residue distances may range from a few to dozens Ångstroms. Naively minimizing the *L*_2_ distance would therefore focused on the large scale structure of the protein and might neglect the small scale structures as they would contribute remarkably less to the loss function. This motivated previous studies to use a threshold value and ignore distances larger than that threshold. For example, AlphaFold (Senior et al., 2020) and RGN (AlQuraishi, 2019a) use a 22.8Å cutoff, which correspond to six times the distance between consecutive *Cα* atoms. During the training phase, we used a slightly more conservative value of 7 residues, which translates to 7 × 3.8Å = 26.6Å. For evaluation and comparison purposes, we report our results using the same cutoff distance of 22.8 Å as RGN.

#### 2.5.4 Regularization

The last component in our optimization scheme is the regularization, *R*(***θ***). We rewrite ***θ*** = [***θ***_0_, … , ***θ***_*L*_] where ***θ***_*j*_ are the parameters used for the *j*-th layer. Then, smooth dynamics are obtained if the total variation of the dynamical system’s parameters is small (Ruthotto and Haber, 2019). Thus we choose the following regularization function

$$R\left(\theta \right)=\underset{j}{\sum }|\theta_{j+1}-\theta_{j}{|}_{1}.$$ (2.17)

**FIGURE 4:**
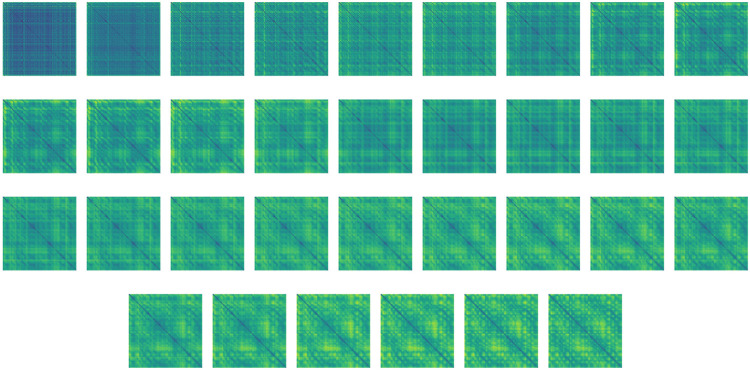
Distance maps of the high-dimensional feature evolution from unstructured (top left) to structured (bottom right) throughout the bi-directional network, during structure prediction of T0783. For a better demonstration of the process smoothness, the network includes 32 layers instead of the six layers used throughout the numerical study. Step size was adjusted to match the integration time. The idea of layer interpolation is discussed in Ruthotto and Haber (2019); Chen et al. (2018); Han et al. (2018).

We note, that since the parameters ***θ***_*i*_ denote the weights of the *i*-th UNet in our network, our regularization term considers the correspondingly-sized layers across subsequent layers of UNets. An example of the rather smooth folding process throughout the network is given in Figure 4. Note that we do not use the standard Tikhonov regularization (so called weight decay) on the weights as they do not guarantee smoothness in time which is crucial for reversible networks and integration in time (Celledoni et al., 2020).

### 2.6 Full Atom Model Reconstruction

**FIGURE 5:**
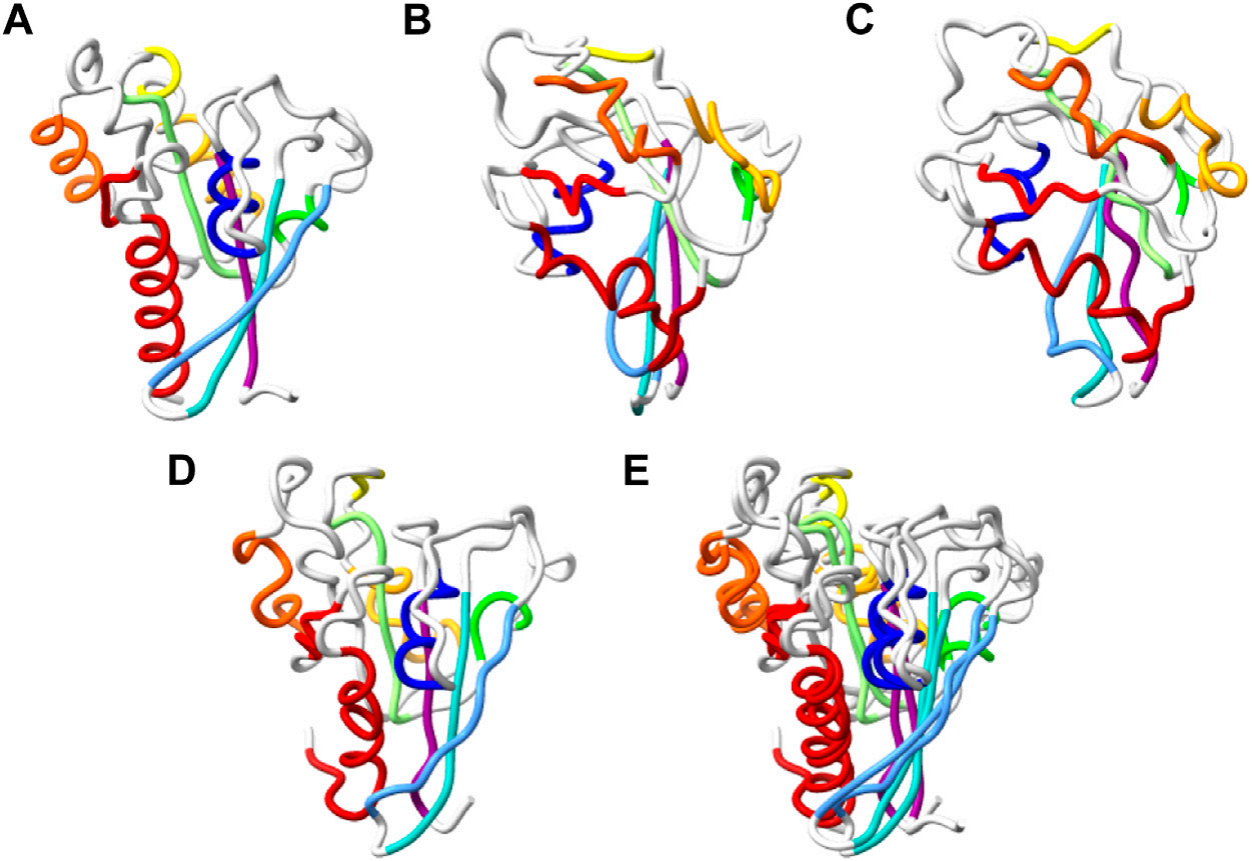
Reconstruction of chirally accurate full atom model. **(A–D)**. Alpha trace representation of: the native structure of T0798 **(A)**, MimNet output with dRMSD 5.194Å, GDTTS 0.19, LDDT 0.424 **(B)**, and two energy minimize models of the MimNet output **(C)** and its mirror image **(D)**. The secondary structure elements of the native structure are colored from first (dark blue) to last (dark red). The atoms of the other models are colored the same, regardless of their conformation. The energy of the minimized model **(C)** is −7838 (arbitrary units) and a dRMSD/GDTTS/LDDT of 5.076Å/0.20/0.426, while the energy of the minimized mirror image **(D)** is −18474, and a dRMSD/GDTTS/LDDT of 4.866Å/0.57/0.4358. **(E)**. A ribbon representation of minimized mirror image, superimposed on the native structure.

While *C*_*α*_ models of protein structures are simple and allow fast structure estimate, they are not useful in practice. This is because it is possible to obtain a low dRMSD score for models that are not physically feasible. Such models can have too short distances between non-consecutive residues, and their geometry may be inverted as the loss function is invariant to mirror symmetry. While coping with this task in an end-to-end fashion is our long-term goal, we currently cope with it with a post processing stage based on our in-house molecular modeling Package MESHI (Kalisman et al., 2005) and scwrl4 (Krivov et al., 2009). An example of a post-processed reconstruction appears in Figure 5. This reconstruction stage consists of four steps:

• Random assignment of *C*_*β*_ and backbone atoms close to their corresponding *C*_*α*_ atoms
• Several rounds of energy minimization, gradually reducing constraint on the *C*_*α*_ atom positions. The energy function includes standard bonded (bond, angle, plane out-of-plane, and torsion-angle (Amir et al., 2008) pairwise non-bonded term (Summa and Levitt, 2007) and hydrogen-bonds terms (Levy-Moonshine et al., 2009).
• Assigning side-chain atoms to their most common rotamer
• Side-chain repacking with scwrl4.

## 3 Numerical Experiments

We verify our method by performing three sets of experiments—protein folding, design, and an ablation study to quantify the contribution of the reversible learning.

### 3.1 Dataset and Settings

#### 3.1.1 Dataset

For the experiments we used the data set supplied by ProteinNet (AlQuraishi, 2019b). The data contains proteins processed from the PDB data set, and is organized to hold training, validation and testing splits specifically for CASP 7–12. The ProteinNet data for CASP 11, for instance, contains 42,338 proteins that are less than 1,000 residue long for training, 224 proteins for validation and 81 test proteins. All training and validation proteins were publicly available before CASP11, and the test set proteins are CASP11 targets. This data set was used in (AlQuraishi, 2019a) and more recently in (Drori et al., 2019). We use the 90% thinning version of the data, as reported in (AlQuraishi, 2019a). While the first order statistics is available, second order statistics cannot be downloaded freely and requires complex and expensive pre-processing. We therefore use only first order statistics in this work. We compare the results to two other recent methods RGN, which uses identical information, and proteinSolver. Note that the recent success of the AlphaFold2, as well as other methods, in CASP 14 were achieved using second order statistics.

#### 3.1.2 Network and Optimization Settings

Throughout our experiments, we use our MimNet as described in Section 2.4 with *n*_*f*_ = 128 with *n*_*Levels*_ = 3 and *T* = 6. Other hyper-parameters, e.g., deeper networks using *T* > 6, may yield better performance and are worthy of consideration at corresponding computational cost. We use the Adam optimizer with a learning rate of 0.0001 and a batch size of 1, trained for 250 epochs. For the protein folding loss described in Eq. 2.5.3, we impose a dynamic mask *M* such that it begins from considering all distances, and linearly decreasing to the cutoff distance used of our training phase, which is 26.6Å. This way, our optimization scheme starts by capturing the global structure of the protein and gradually optimizing local interactions (distances) between residues. Our experiments are carried on an Nvidia Titan RTX. Our code is implemented in PyTorch (Paszke et al., 2019).

#### 3.1.3 Comparisons

To evaluate our results we compare them to two recent studies, in the fields of protein design and PSP. First, for protein design, we compare our results with those of (Strokach et al., 2020). Their ProteinSolver is a graph neural network for the solution of the design problem. Using a sophisticated graph representation of the protein and its features, ProteinSolver obtains state of the art results, and a remarkable improvement over previous design studies. Thus, we consider it a good benchmark. Second, the results of the PSP side of our study are compared to the work of (AlQuraishi, 2019a). While that work did not achieve state of the art results (compared with methods that use second-order statistics), it is the only recent PSP study, known to us, that uses first order information from the ProteinNet dataset only.

### 3.2 Protein Design

The design experiments used *C*_*α*_ models, obtained from ProteinNet, to predict the corresponding PSSMs. As discussed in Section 2.5.2, the PSSMs are soft-assignments of the designed sequences, in accordance with the multiple design possibilities given a structure. The KL-divergence between the predicted PSSM and the ground-truth PSSM as supplied by the data in proteinNet is thus a natural measure, which we use for both training and performance evaluation. To compare our results with the recent work of ProteinSolver (Strokach et al., 2020) we employed the code and weights that are supplied with the paper (based on the 2015 version of the PDB) and used this pre-trained model to generate PSSMs for the proteinNet test sets data (CASP 7–12). The PSSMs predicted by ProteinSolver are then compared to the true PSSMs using KL-divergence. Table 1 depicts the design performances of proteinSolver and our MimNet on the various ProteinNet test sets. It shows a major improvement in MimNet, although ProteinSolver utilizes a superset of both the training and test sets of (CASP 7–11).

**TABLE 1:** KL-divergence comparison of recent Protein-Design methods.

Model	CASP 7	CASP 8	CASP 9	CASP 10	CASP 11	CASP 12
ProteinSolver Strokach et al. (2020)	1.73	1.61	1.63	1.5	1.67	1.62
MimNet (ours)	0.98	0.87	0.92	0.88	0.91	0.94

### 3.3 Protein Folding

Our folding experiment used first-order data (PSSM and a one hot sequence encoding), obtained from the ProteinNet test sets, to generate *C*_*α*_ models of the corresponding native structures. The accuracies of these models are evaluated by dRMSD to make the results comparable to those of RGN that used the same data sets (AlQuraishi, 2019a). Table 2 depicts the average performances of each method on the different test sets, suggest that the use of MimNet can significantly improve the accuracy of protein folding.

**TABLE 2:** dRMSD (Å) comparison of recent Protein-Folding methods. Averages of FM (novel folds)/TBM (known folds) are shown.

Model	CASP 7	CASP 8	CASP 9	CASP 10	CASP 11	CASP 12
RGN AlQuraishi, (2019a)	9.3/5.6	7.3/5.9	8.7/6.5	10.0/6.9	8.5/7.4	10.7/6.9
MimNet (ours)	5.8/5.4	6.1/5.5	6.4/5.8	6.6/6.5	6.6/5.6	6.4/6.0

### 3.4 Additional Experimental Setup

**FIGURE 6:**
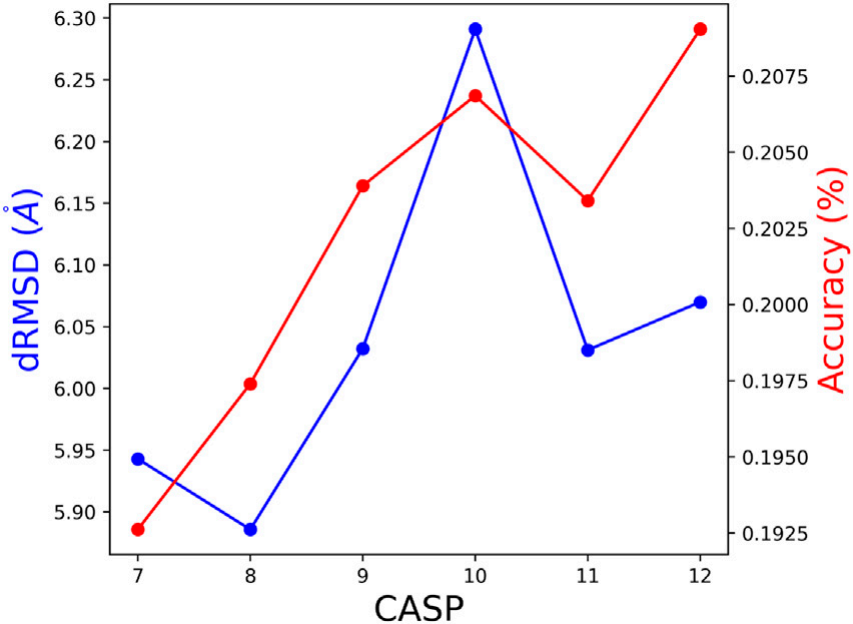
The obtained dRMSD and residue accuracy on the union of test of subsequent CASP editions. (e.g., the test set for CASP 8 is a union of CASP 8 until 12).

To further test the method’s performance, we deviated from the ProteinNet scheme by creating new test sets. To this end, performance of a model based on CASP_*i*_ training set, is tested by the union of all CASP test sets CASP*i*, … ,CASP12. This way, all but the last model were tested on larger and somewhat different test sets. The performances, presented in Figure 6, are consistent with results of the standard test sets reported in Tables 2, 4. We show the dRMSD and Accuracy, which is consistent with the KL divergence score.

### 3.5 Per-Target Performance

**FIGURE 7:**
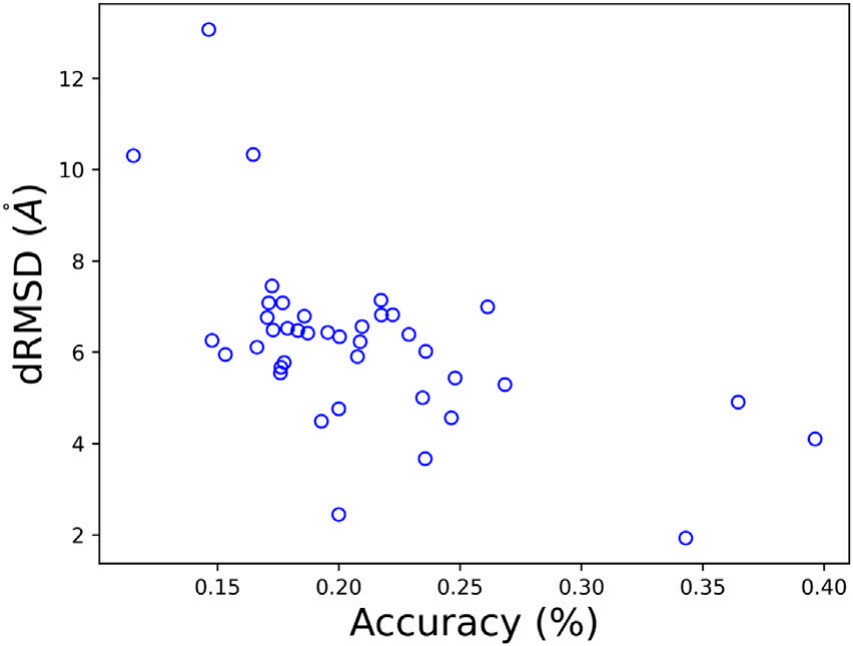
A comparison of the obtained dRMSD and residue accuracy on CASP 12 test set with our MimNet.

Per-target performance in both folding and design is presented in Figure 7, and is consistent with the average values presented the Table 3. Interestingly, we can see a negative correlation between the obtained dRMSD and accuracy. That is, where our network yields lower (better) dRMSD, a higher sequence prediction accuracy is obtained.

**TABLE 3:** A comparison of the reversible and standard ResNet architectures on folding and design tasks on CASP 11. For the folding task, we report both FM and TBM proteins dRMSD (Å) scores. For the design task, the KL-divergence of the PSSM and sequence (one-hot labels) are reported, along with the sequence prediction accuracy.

Model	Folding		Design	
	FM	TBM	PSSM	One-hot
MimNet (ResNet)	7.1	5.9	1.00	1.77 (19.9%)
MimNet (Reversible)	6.6	5.6	0.93	1.73 (20.7%)

### 3.6 The Significance of Reversibility

One of the key contributions of our work is the introduction of reversible network architecture that can jointly learn both protein folding and design, which have not been done until now. We therefore delve on the significance of the reversibility scheme. There are three advantages for a reversible network architecture over a one directional network. First, and most important, typical one directional graph networks (e.g., ResNet like) are known to have smoothing properties that do not recover high frequencies well (Ming Chen et al., 2020; Zhao and Akoglu, 2020), a problem that can be alleviated by using second-order dynamics networks (Eliasof et al., 2021), which are also reversible, and behave like our dynamics in 2.3. Second, although we have not used this property in this work, reversible network allow to use very deep networks without limitation on memory (Gomez et al., 2017). Finally, in some cases, reversible architecture yields better results in training and generalizing compared to their one directional counterparts (Zhu et al., 2017; Chang et al., 2018; Yang et al., 2019). Most importantly it is a common approach in Language models (Devlin et al., 2018).

To validate the observations above on the considered data-set, we compare the reversible architecture to a standard ResNet (as in Eq. 2.4) architecture using the same settings. Namely, we employ the same input and output as well as number of layers and channels (as described in Section 3.1.2), yielding an identical number of parameters. The results for this experiment are provided in Table 3. The results suggest that using the reversible architecture is favored.

We now examine the importance of training the network in a bi-directional manner. We compare the behavior of our network when trained for both directions, versus the case of optimizing it only one direction (from sequence to coordinates and vice versa). Our results are summarized in Tables 4, 5 , suggesting that in some cases coupling the learning of folding and design problems can lead to better results in both folding and design, however, the effect of training on both sets of data at the same time, was marginal. Curiously, the reversible training experiments were our original motivation for the inclusion of PSSM in the design loss function (for symmetry considerations). In retrospect it turned out that this has a considerable positive effect on the design performance. Notably, the accuracy of *one-hot* prediction improves when the network is trained using PSSM (Table 4).

**TABLE 4:** Comparison between Coordinates to Design (C → D) and reversible learning (C ↔ D) on CASP 7–12. The letters in the brackets represent the data considered by the loss function. P denotes PSSM, O denotes one-hot encoding of the sequence and P/O implies both data are utilized. We report the KL-divergence scores of the PSSM, the sequence or both. The accuracy of the sequence is depicted in brackets where applicable.

Dataset	C → D (P)	C → D (O)	C → D (P/O)	C ↔ D(P)	C ↔ D(P/O)
CASP 7	1.08	1.79 (16.7%)	1.02/1.69 (23.2%)	0.98	0.96/1.69 (23.1%)
CASP 8	0.87	1.77 (14.2%)	0.89/1.67 (21.1%)	0.87	0.88/1.64 (21.5%)
CASP 9	0.90	1.74 (13.9%)	0.90/1.61 (21.3%)	0.92	0.91/1.62 (21.0%)
CASP 10	0.83	1.76 (16.1%)	0.84/1.65 (21.7%)	0.88	0.83/1.69 (21.2%)
CASP 11	0.98	1.82 (14.3%)	1.00/1.75 (20.2%)	0.91	0.93/1.73 (20.7%)
CASP 12	0.97	1.85 (14.0%)	0.99/1.75 (20.3%)	0.94	0.91/1.74 (20.3%)

**TABLE 5:** Comparison between Design to Coordinates (D → C) and reversible learning (C ↔ D) on CASP 7–12. Results are reported in dRMSD (Å).

Dataset	D → C	D ↔ C
CASP 7	5.41	5.50
CASP 8	5.54	5.58
CASP 9	5.89	5.83
CASP 10	6.86	6.61
CASP 11	5.91	5.84
CASP 12	6.32	6.07

## 4 Conclusion

In this work we introduce a novel approach that unifies the treatment of protein folding and protein design. Our methodology is based on a combination of two recently studied techniques developed in deep learning. The first is a reversible architecture. Such an architecture allows us to propagate forward and backward and therefore have a network that can propagate sequence information into coordinates information and, more importantly for the protein design, propagate backward from a structure to a sequence. In the context of employing neural networks for molecular dynamics, it is natural to couple the reversible architecture with a graph representation, since our network models the pairwise interactions between amino acids as edges in the graph. Furthermore, to allow far field interactions, a multiscale structure is used.

In this scheme the input of one direction is the output of the other. The folding direction starts from a sequence and a PSSM formulated as a matrix of residue probabilities, and its output is a protein conformation. The loss function for training is the standard dRMSD metric applied to the output conformation and the native structure. Beyond the scope of this study, the scores of general PSSMs may not represent probabilities. In such cases however one can employ the SoftMax scheme to convert the scores to residue probabilities. The native structure in turn is the input to the design direction and the output is a PSSM and sequence, with their KL-divergence serving as the loss function for training. Previous studies only derived a single output from the PSSM. We note that the design problem does not have a unique answer and therefore we follow a more probabilistic approach.

We have performed extensive numerical experiments that compares both folding and design on the CASP 7–12 data sets. These data sets contain tens of thousands of proteins that we trained both on folding and design tasks. We compared the results of the protein folding to a recent work that uses only first order information. We have shown that our network performs on par or better than that network for the folding task. More importantly however, we have shown a significant improvement on the protein design task, achieving a KL-divergence loss that is less than half of a recently published work. We attribute this success to the use of recent protein folding architectures as well as using extensive data sets that allow better training of the proposed architecture. We note that our network is generic and can use both first order statistics, as in this study, and second order statistics.

Finally, we believe that the ProteinNet data-set constitutes a great leap forward as it allows scientists to compare methods on the same footings, similarly to the impact of ImageNet (Deng et al., 2009) on the computer vision community.

## Data Availability

Publicly available datasets were analyzed in this study. This data can be found here: ProteinNet at github.com/aqlaboratory/proteinnet.

## References

[B1] Abriata L. A. Tamò G. E. Dal Peraro M. (2019). A Further Leap of Improvement in Tertiary Structure Prediction in Casp13 Prompts New Routes for Future Assessments. Proteins 87, 1100–1112. 10.1002/prot.25787 31344267

[B2] Adamczak R. Porollo A. Meller J. (2004). Accurate Prediction of Solvent Accessibility Using Neural Networks-Based Regression. Proteins 56, 753–767. 10.1002/prot.2017 15281128

[B3] Allen M. P. (2004). Introduction to Molecular Dynamics Simulation. Comput. soft matter: Synth. Polym. proteins 23, 1–28.

[B4] AlQuraishi M. (2019a). End-to-end Differentiable Learning of Protein Structure. Cell Syst 8 (4), 292–e3. 10.1016/j.cels.2019.03.006 31005579 PMC6513320

[B5] AlQuraishi M. (2019b). Proteinnet: a Standardized Data Set for Machine Learning of Protein Structure. BMC Bioinformatics 20, 311. 10.1186/s12859-019-2932-0 31185886 PMC6560865

[B6] Altschul S. F. Madden T. L. Schäffer A. A. Zhang J. Zhang Z. Miller W. (1997). Gapped BLAST and PSI-BLAST: a New Generation of Protein Database Search Programs. Nucleic Acids Res. 25, 3389–3402. 10.1093/nar/25.17.3389 9254694 PMC146917

[B7] Amir E. D. Kalisman N. Keasar C. (2008). Differentiable, Multi-Dimensional, Knowledge-Based Energy Terms for Torsion Angle Probabilities and Propensities. Proteins 72, 62–73. 10.1002/prot.21896 18186478

[B8] Anand-Achim N. Eguchi R. R. Mathews I. I. Perez C. P. Derry A. Altman R. B. (2021). Protein Sequence Design with a Learned Potential. bioRxiv. 10.1101/2020.01.06.895466 PMC8826426 35136054

[B9] Baek M. DiMaio F. Anishchenko I. Dauparas J. Ovchinnikov S. Lee G. R. (2021). Accurate Prediction of Protein Structures and Interactions Using a Three-Track Neural Network. Science 373, 871–876. 10.1126/science.abj8754 34282049 PMC7612213

[B10] Basanta B. Bick M. J. Bera A. K. Norn C. Chow C. M. Carter L. P. (2020). An Enumerative Algorithm for De Novo Design of Proteins with Diverse Pocket Structures. Proc. Natl. Acad. Sci. U S A. 117, 22135–22145. 10.1073/pnas.2005412117 32839327 PMC7486743

[B11] Bates P. A. Kelley L. A. MacCallum R. M. Sternberg M. J. (2001). Enhancement of Protein Modeling by Human Intervention in Applying the Automatic Programs 3d-Jigsaw and 3d-Pssm. Proteins 5, 39–46. 10.1002/prot.1168 11835480

[B12] Ben-Sasson A. J. Watson J. L. Sheffler W. Johnson M. C. Bittleston A. Somasundaram L. (2021).Design of Biologically Active Binary Protein 2D Materials. Nature 589. 468–473. 10.1038/s41586-020-03120-8 33408408 PMC7855610

[B13] Bershtein S. Goldin K. Tawfik D. S. (2008). Intense Neutral Drifts Yield Robust and Evolvable Consensus Proteins. J. Mol. Biol. 379, 1029–1044. 10.1016/j.jmb.2008.04.024 18495157

[B14] Bouritsas G. Bokhnyak S. Ploumpis S. Bronstein M. Zafeiriou S. (2019). “Neural 3d Morphable Models: Spiral Convolutional Networks for 3d Shape Representation Learning and Generation,” in Proceedings of the IEEE/CVF International Conference on Computer Vision, 7213–7222. 10.1109/iccv.2019.00731

[B15] Celledoni E. Ehrhardt M. J. Etmann C. McLachlan R. I. Owren B. Schönlieb C.-B. (2020). Structure Preserving Deep Learning. *arXiv preprint arXiv:2006.03364* .

[B16] Chamberlain B. P. Rowbottom J. Gorinova M. Webb S. Rossi E. Bronstein M. M. (2021). Grand: Graph Neural Diffusion. *arXiv preprint arXiv:2106.10934* .

[B17] Chandler P. G. Broendum S. S. Riley B. T. Spence M. A. Jackson C. J. McGowan S. (2020). Strategies for Increasing Protein Stability. New York, NY: Springer US, 163–181. 10.1007/978-1-4939-9869-2_10 31612442

[B18] Chang B. Meng L. Haber E. Ruthotto L. Begert D. Holtham E. (2018). Reversible Architectures for Arbitrarily Deep Residual Neural Networks. In Proceedings of the AAAI Conference on Artificial Intelligence, 32.

[B19] Chen R. T. Q. Rubanova Y. Bettencourt J. Duvenaud D. (2018). Neural Ordinary Differential Equations. *32nd Conference On Neural Information Processing Systems (NeurIPS)* .

[B20] Dahiyat B. I. Mayo S. L. (1996). Protein Design Automation. Protein Sci. 5, 895–903. 10.1002/pro.5560050511 8732761 PMC2143401

[B21] Deng J. Dong W. Socher R. Li L.-J. Li K. Fei-Fei L. (2009). “ImageNet: A Large-Scale Hierarchical Image Database,” in CVPR09. 10.1109/cvpr.2009.5206848

[B22] Devlin J. Chang M.-W. Lee K. Toutanova K. (2018). Bert: Pre-training of Deep Bidirectional Transformers for Language Understanding. *arXiv preprint arXiv:1810.04805* .

[B23] Drori I. Thaker D. Srivatsa A. Jeong D. Wang Y. Nan L. (2019). Accurate Protein Structure Prediction by Embeddings and Deep Learning Representations. Machine Learn. Comput. Biol. (Mlcb).

[B24] Eliasof M. Haber E. Treister E. (2021). Pde-gcn: Novel Architectures for Graph Neural Networks Motivated by Partial Differential Equations. Adv. Neural Inf. Process. Syst. 34.

[B25] Eliasof M. Treister E. (2020). Diffgcn: Graph Convolutional Networks via Differential Operators and Algebraic Multigrid Pooling. *34th Conference On Neural Information Processing Systems (NeurIPS 2020)* . Vancouver, Canada.

[B26] Gao H. Ji S. (2019). “Graph U-Nets,” in international conference on machine learning (PMLR), 2083–2092.

[B27] Gao Y. Wang S. Deng M. Xu J. (2018). Raptorx-angle: Real-Value Prediction of Protein Backbone Dihedral Angles through a Hybrid Method of Clustering and Deep Learning. BMC Bioinformatics 19, 100. 10.1186/s12859-018-2065-x 29745828 PMC5998898

[B28] Gomez A. N. Ren M. Urtasun R. Grosse R. B. (2017). “The Reversible Residual Network: Backpropagation without Storing Activations,” in Proceedings of the 31st International Conference on Neural Information Processing Systems, 2211–2221.

[B29] Goodfellow I. Bengio Y. Courville A. (2016). Deep Learning. MIT Press. Available at: http://www.deeplearningbook.org .

[B30] Gribskov M. McLachlan A. D. Eisenberg D. (1987). Profile Analysis: Detection of Distantly Related Proteins. Proc. Natl. Acad. Sci. U S A. 84, 4355–4358. 10.1073/pnas.84.13.4355 3474607 PMC305087

[B31] Haas J. Barbato A. Behringer D. Studer G. Roth S. Bertoni M. (2018). Continuous Automated Model Evaluation (Cameo) Complementing the Critical Assessment of Structure Prediction in Casp12. Proteins 86, 387–398. 10.1002/prot.25431 29178137 PMC5820194

[B32] Han J. Jentzen A. E W. (2018). Solving High-Dimensional Partial Differential Equations Using Deep Learning. Proc. Natl. Acad. Sci. U S A. 115, 8505–8510. 10.1073/pnas.1718942115 30082389 PMC6112690

[B33] Hanocka R. Hertz A. Fish N. Giryes R. Fleishman S. Cohen-Or D. (2019). MeshCNN. ACM Trans. Graph. 38, 1–12. 10.1145/3306346.3322959

[B34] He K. Zhang X. Ren S. Sun J. (2016). “Deep Residual Learning for Image Recognition,” in Proceedings of the IEEE Conference on Computer Vision and Pattern Recognition, 770–778. 10.1109/cvpr.2016.90

[B35] Hildebrand A. Remmert M. Biegert A. Söding J. (2009). Fast and Accurate Automatic Structure Prediction with HHpred. Proteins 77, 128–132. 10.1002/prot.22499 19626712

[B36] Hou J. Wu T. Cao R. Cheng J. (2019). Protein Tertiary Structure Modeling Driven by Deep Learning and Contact Distance Prediction in Casp13. Proteins 87, 1165–1178. 10.1002/prot.25697 30985027 PMC6800999

[B37] Ingraham J. Garg V. K. Barzilay R. Jaakkola T. (2019). “Generative Models for Graph-Based Protein Design,” in Advances in Neural Information Processing Systems.

[B38] Jones D. T. Buchan D. W. Cozzetto D. Pontil M. (2012). PSICOV: Precise Structural Contact Prediction Using Sparse Inverse Covariance Estimation on Large Multiple Sequence Alignments. Bioinformatics 28, 184–190. 10.1093/bioinformatics/btr638 22101153

[B39] Jones D. T. (1994). De Novo protein Design Using Pairwise Potentials and a Genetic Algorithm. Protein Sci. 3, 567–574. 10.1002/pro.5560030405 8003975 PMC2142856

[B40] Jumper J. Evans R. Pritzel A. Green T. Figurnov M. Ronneberger O. (2021). Highly Accurate Protein Structure Prediction with Alphafold. Nature, 1–11. 10.1038/s41586-021-03819-2 PMC8371605 34265844

[B41] Kalisman N. Levi A. Maximova T. Reshef D. Zafriri-Lynn S. Gleyzer Y. (2005). MESHI: a New Library of Java Classes for Molecular Modeling. Bioinformatics 21, 3931–3932. 10.1093/bioinformatics/bti630 16105898

[B42] Kamisetty H. Ovchinnikov S. Baker D. (2013). Assessing the Utility of Coevolution-Based Residue-Residue Contact Predictions in a Sequence- and Structure-Rich Era. Proc. Natl. Acad. Sci. U S A. 110, 15674–15679. 10.1073/pnas.1314045110 24009338 PMC3785744

[B43] Kandathil S. M. Greener J. G. Jones D. T. (2019). Recent Developments in Deep Learning Applied to Protein Structure Prediction. Proteins 87, 1179–1189. 10.1002/prot.25824 31589782 PMC6899861

[B44] Krivov G. G. Shapovalov M. V. Dunbrack R. L. (2009). Improved Prediction of Protein Side-Chain Conformations with SCWRL4. Proteins 77, 778–795. 10.1002/prot.22488 19603484 PMC2885146

[B45] Krizhevsky A. Sutskever I. Hinton G. E. (2012). “Imagenet Classification with Deep Convolutional Neural Networks,” in Advances in neural information processing systems, 1097–1105.

[B46] Kryshtafovych A. Schwede T. Topf M. Fidelis K. Moult J. (2019). Critical Assessment of Methods of Protein Structure Prediction (CASP)-Round XIII. Proteins 87, 1011–1020. 10.1002/prot.25823 31589781 PMC6927249

[B47] Leaver-Fay A. Tyka M. Lewis S. M. Lange O. F. Thompson J. Jacak R. (2011). “Rosetta3,” in Methods in Enzymology. Editors Johnson M. L. Brand L. (Academic Press), 487, 545–574. of Computer Methods, Part C. 10.1016/B978-0-12-381270-4.00019-6 PMC4083816 21187238

[B48] Levy-Moonshine A. Amir el-A. D. Keasar C. (2009). Enhancement of Beta-Sheet Assembly by Cooperative Hydrogen Bonds Potential. Bioinformatics 25, 2639–2645. 10.1093/bioinformatics/btp449 19628506 PMC3140807

[B49] Li H. Hou J. Adhikari B. Lyu Q. Cheng J. (2017). Deep Learning Methods for Protein Torsion Angle Prediction. BMC Bioinformatics 18, 417–426. 10.1186/s12859-017-1834-2 28923002 PMC5604354

[B50] Li Y. Hu J. Zhang C. Yu D. J. Zhang Y. (2019). ResPRE: High-Accuracy Protein Contact Prediction by Coupling Precision Matrix with Deep Residual Neural Networks. Bioinformatics 35, 4647–4655. 10.1093/bioinformatics/btz291 31070716 PMC6853658

[B51] Ming Chen Z. W. Zengfeng Huang B. D. Li Y. (2020). Simple and Deep Graph Convolutional Networks.

[B52] Morcos F. Pagnani A. Lunt B. Bertolino A. Marks D. S. Sander C. (2011). Direct-coupling Analysis of Residue Coevolution Captures Native Contacts across many Protein Families. Proc. Natl. Acad. Sci. U S A. 108, E1293–E1301. 10.1073/pnas.1111471108 22106262 PMC3241805

[B53] Moult J. Hubbard T. Bryant S. H. Fidelis K. Pedersen J. T. (1997). Critical Assessment of Methods of Protein Structure Prediction (Casp): Round Ii. Proteins Suppl 1, 2–6. 10.100210.1002/(sici)1097-0134(1997)1+<2::aid-prot2>3.0.co;2-t 9485489

[B54] Norn C. Wicky B. I. M. Juergens D. Liu S. Kim D. Tischer D. (2021). Protein Sequence Design by Conformational Landscape Optimization. Proc. Natl. Acad. Sci. 118. 10.1073/pnas.2017228118 PMC7980421 33712545

[B55] O'Connell J. Li Z. Hanson J. Heffernan R. Lyons J. Paliwal K. (2018). Spin2: Predicting Sequence Profiles from Protein Structures Using Deep Neural Networks. Proteins 86, 629–633. 10.1002/prot.25489 29508448

[B56] Paszke A. Gross S. Massa F. Lerer A. Bradbury J. Chanan G. (2019). “Pytorch: An Imperative Style, High-Performance Deep Learning Library,” in Advances in Neural Information Processing Systems 32. Editors Wallach H. Larochelle H. Beygelzimer A. d'Alché-Buc F. Fox E. Garnett R. (Curran Associates, Inc.), 8024–8035.

[B57] Ranjan A. Bolkart T. Sanyal S. Black M. J. (2018). “Generating 3d Faces Using Convolutional Mesh Autoencoders,” in Proceedings of the European Conference on Computer Vision (ECCV), 704–720. 10.1007/978-3-030-01219-9_43

[B58] Remmert M. Biegert A. Hauser A. Söding J. (2012). HHblits: Lightning-Fast Iterative Protein Sequence Searching by HMM-HMM Alignment. Nat. Methods 9, 173–175. 10.1038/nmeth.1818 22198341

[B59] Ronneberger O. Fischer P. Brox T. (2015). U-net: Convolutional Networks for Biomedical Image Segmentation.

[B60] Rost B. Casadio R. Fariselli P. Sander C. (1995). Transmembrane Helices Predicted at 95% Accuracy. Protein Sci. 4, 521–533. 10.1002/pro.5560040318 7795533 PMC2143072

[B61] Rost B. Sander C. (1993). Prediction of Protein Secondary Structure at Better Than 70% Accuracy. J. Mol. Biol. 232, 584–599. 10.1006/jmbi.1993.1413 8345525

[B62] Ruthotto L. Haber E. (2019). Deep Neural Networks Motivated by Partial Differential Equations. J. Math. Imaging Vis., 1–13. 10.1007/s10851-019-00903-1

[B63] Schwede T. Kopp J. Guex N. Peitsch M. C. (2003). SWISS-MODEL: an Automated Protein Homology-Modeling Server. Nucleic Acids Res. 31, 3381–3385. 10.1093/nar/gkg520 12824332 PMC168927

[B64] Senior A. W. Evans R. Jumper J. Kirkpatrick J. Sifre L. Green T. (2020). Improved Protein Structure Prediction Using Potentials from Deep Learning. Nature 577, 706–710. 10.1038/s41586-019-1923-7 31942072

[B65] Shah S. Ghosh P. Davis L. S. Goldstein T. (2018). Stacked U-Nets: A No-Frills Approach to Natural Image Segmentation

[B66] Silverman B. W. (1986). Density Estimation for Statistics and Data Analysis. London: Chapman & Hall.

[B67] Strokach A. Becerra D. Corbi-Verge C. Perez-Riba A. Kim P. M. (2020). Fast and Flexible Protein Design Using Deep Graph Neural Networks. Cel Syst 11, 402–e4. 10.1016/j.cels.2020.08.016 32971019

[B68] Summa C. M. Levitt M. (2007). Near-native Structure Refinement Using In Vacuo Energy Minimization. Proc. Natl. Acad. Sci. U S A. 104, 3177–3182. 10.1073/pnas.0611593104 17360625 PMC1802011

[B69] Sun M. G. F. Kim P. M. (2017). Data Driven Flexible Backbone Protein Design. Plos Comput. Biol. 13, e1005722. 10.1371/journal.pcbi.1005722 28837553 PMC5587332

[B70] Tetchner S. Kosciolek T. Jones D. T. (2014). Opportunities and Limitations in Applying Coevolution-Derived Contacts to Protein Structure Prediction. Bio-Algorithms and Med-Systems 10, 243–254. 10.1515/bams-2014-0013

[B71] Torrisi M. Pollastri G. Le Q. (2020). Deep Learning Methods in Protein Structure Prediction. Comput. Struct. Biotechnol. J. 18, 1301–1310. 10.1016/j.csbj.2019.12.011 32612753 PMC7305407

[B72] Vassura M. Margara L. Di Lena P. Medri F. Fariselli P. Casadio R. (2008). Reconstruction of 3d Structures from Protein Contact Maps. Ieee/acm Trans. Comput. Biol. Bioinform 5, 357–367. 10.1109/TCBB.2008.27 18670040

[B73] Wang Y. Sun Y. Liu Z. Sarma S. E. Bronstein M. M. Solomon J. M. (2019). Dynamic Graph Cnn for Learning on point Clouds. ACM Trans. Graph. 38, 1–12. 10.1145/3326362

[B74] Waterhouse A. Bertoni M. Bienert S. Studer G. Tauriello G. Gumienny R. (2018). SWISS-MODEL: Homology Modelling of Protein Structures and Complexes. Nucleic Acids Res. 46, W296–W303. 10.1093/nar/gky427 29788355 PMC6030848

[B75] Xu J. (2019). Distance-based Protein Folding Powered by Deep Learning. Proc. Natl. Acad. Sci. U S A. 116, 16856–16865. 10.1073/pnas.1821309116 31399549 PMC6708335

[B76] Xu J. Mcpartlon M. Li J. (2021). Improved Protein Structure Prediction by Deep Learning Irrespective of Co-evolution Information. Nat. Machine Intelligence 1–9. 10.1038/s42256-021-00348-5 PMC8340610 34368623

[B77] Yang G. Huang X. Hao Z. Liu M.-Y. Belongie S. Hariharan B. (2019). Pointflow: 3d point Cloud Generation with Continuous Normalizing Flows.

[B78] Zhao L. Akoglu L. (2020). “Pairnorm: Tackling Oversmoothing in \{gnn\}s,” in International Conference on Learning Representations.

[B79] Zheng W. Li Y. Zhang C. Pearce R. Mortuza S. M. Zhang Y. (2019). Deep-learning Contact-Map Guided Protein Structure Prediction in Casp13. Proteins 87, 1149–1164. 10.1002/prot.25792 31365149 PMC6851476

[B80] Zhu J.-Y. Park T. Isola P. Efros A. A. (2017). “Unpaired Image-To-Image Translation Using Cycle-Consistent Adversarial Networks,” in Proceedings of the IEEE international conference on computer vision, 2223–2232. 10.1109/iccv.2017.244

